# Empowerment of the Rural Parents/Caregivers of Children with Heart Diseases in Namibia to Facilitate Coping with the Demands of Caring at Home

**DOI:** 10.5539/gjhs.v5n2p74

**Published:** 2012-12-04

**Authors:** Kristofina Amakali, Louis F. Small

**Affiliations:** 1 School of Nursing and Public Health, University of Namibia (UNAM), Windhoek, Namibia

**Keywords:** parents, caregivers, empowerment, coping, care, children, heart diseases, home-based care

## Abstract

**Aim:**

The purpose of this study was to describe how the parents/caregivers of children with heart diseases cope with the demands of caring for these children at home, with the purpose to develop a home-based health care programme to facilitate the parents/caregivers’ coping with the demands of care.

**Methods:**

A qualitative, exploratory, descriptive, and contextual study was conducted. Phenomenological data on the lived experiences of coping with the demands to care by the parents/caregivers and of living with the burden of the disease by the children were gathered and interpreted from a purposefully selected sample of 5 multiple cases of parents/caregivers and children with heart disease from the rural areas.

**Results:**

The findings have revealed poor coping with the demands of caring among the parents/caregivers, as characterized by the experiences of emotional challenges, disruptive social functioning and social relations, lack of support from the family and societal organizations, financial difficulties and of course the experiences of decreased vitality by the children. As a result, the need to empower the parents/caregivers for them to cope with providing a continuum care to their children who have heart disease was identified. The dynamics to mitigate the negative experiences were conceptualized. Therefore, the interventions of a home-based health care programme as an interface to facilitate the parents/caregivers to cope with the challenges caused by the demands to care were developed.

**Conclusions:**

The need for empowerment of these parents/caregivers can be met through the implementation of multi-component interventions, which draw together all the possible determinants factors and the coping methods to facilitate coping.

## 1. Introduction

Heart diseases interfere with the most basic human requirement, namely, the need for a continuous supply of well oxygenated blood to the body systems. In Namibia, heart diseases contribute to approximately ten percent (10%) of all paediatric admissions to health care facilities (WHO, 2009). Congenital heart defects, mostly the septal defects, patent ductus arteriousus, coarctation of aorta and less Tetralogy of Fallot are the main causes of these heart diseases, accounting for sixty percent (60%), while rheumatic fever (infection) accounts for forty percent (40%) of all paediatric patients with heart disease in Namibia and approximately all (90%) of these children are from the rural areas. Clearly the situation in Namibia is grave because a significant number (close to 500) of children are currently in need of cardiac (heart) surgery for congenital and rheumatic heart diseases.

The diagnosis of a heart disease, the perception of the nature of the disease, its potential future courses and the experiences of related diminished functional status, inevitably causes anxiety for the children. Their parents or caregivers also experience powerlessness to provide care to their loved ones at home (Shu-Fan, Pei-Fan, & Kai-Sheng, 2007). The proponents of health promotion advocate for the empowerment of the caregivers to be able to provide appropriate home-based care to their significant ones who are living with long-term illnesses and therefore for them to cope with the demands of caring (Forster, 2008).

Empowerment is a process of overcoming a sense of powerlessness and a mechanism to assist people to redefine the challenges, to conceptualize them as manageable and to develop inner strength and self-determination (Mitchell, 2011; Raina et al., 2005).

In contrast to emphasizing the limitations and the problems associated with the child's illness, empowerment of the parents and caregivers of children with heart diseases from the rural areas of Namibia includes the identification and utilization of the strengths within the family and at the community level to facilitate coping with the demands of care. Adequate information about the child's condition and the care that is needed, the availability of social support, material resources and financial means can enhance the parents/caregivers’ self-esteem and therefore facilitate the parents and caregivers to cope with the demands of care at home. Thus, the quality of care for the children with heart diseases from the rural area of Namibia is not determined only by their family, but also by the many spheres of influences that can impact on the quality of care that their parents provide (Berkman, 1995).

Thus the philosophy of a home-based health care emphasizes the harnessing of resources and bolstering of optimism for the caregivers to cope with a situation that is viewed as discouraging and hopeless, and therefore to inculcate an attitude which promises positive returns for the children. In addition to facilitating coping with the demands of care, the children with heart diseases can also be empowered to use different coping methods and reconceptualization of the symptoms of the disease as a manageable challenge despite its restrictions (Kettunen, Oskiparta, & Liimatainen, 2001; Hadley, Hair, & Anderson More, 2008).

This study presents a conceptualization of a home-based health care programme of multi-component interventions, which draws together all the possible determinants factors and the coping methods to the Namibian rural parents/caregivers and their children who have heart diseases for the implementation of self-care goal directed activities to facilitate coping with the demands of caring at home, the results of which can reduce the burden of caregiving, enhance coping with the demands of care as well as coping with the disease burden for the children (Gitlin et al., 2003; Acton & Kang, 2001).

### 1.1 Method of Conceptualization of a Home-Based Health Care Programme

The central concept about the findings- the need for support for the parents/caregivers in regard to coping with the demands of caring for their children as well as support to enable the children to adapt to living with a heart disease was identified from the themes that were arrived at in situational analysis of the study. Accordingly, Dickoff et al. (1964)'s concepts of a situation-producing was employed as a framework for the operational description of the envisaged home-based health care programme to facilitate coping with the demands of caring by the parents and coping with the diseases burden by the children.

## 2. A Home-Base Health Care Programme to Facilitate Parents/Caregivers’ Coping with the Demands of Care at Home

In this regard, the home-based health care programme was conceptualized according to some of Dicoff et al. (1964)'s essential ingredients of a situation-producing concept and these include the goal-content, which encompasses the aim of the home-based health care programme and the desired situation to be brought into existence-the facilitation of coping with the demands of care at home, the prescription for activities or the actions to be taken, which are appropriate, and are therefore likely to lead to the realization of the goal content.

The activity prescription includes the concept that while much is expected of a facilitator for the implementation of the home-based health care programme (Webb, 2009; Glanz, Rimer, & Viswanath, 2008; George, 2008; Krapp, 2005; Major, 2003; Pecrun, Goetz, Titz, & Perry, 2002), it is important that the parents/caregivers as the recipients of the empowerment programme should possess the essential characteristics that can enable them to be responsive and receptive towards learning how to provide care for their children who have the heart diseases and for them to cope with the demands of care as a desired outcome. These include but not exclusive to a sense of responsibility, understanding, knowledge, willingness, motivation, confidences or the belief in what one does, self-efficacy or the belief in one's own ability to change the situation and the necessary skills to carry out instrumental tasks of caring to the child (Wåhlin, Ek, & Idvall, 2009; George, 2008; Huberto-Augustin, 2008; Strömberg, 2005; Ågren, 2008; Deyirmenjian, Karam, & Salameh, 2005; Gogineni, 1998). By consequence of empowerment with these characteristics, the parents and caregivers would be competent, with confidence and as a result, they can provide appropriate and safer care to their children who have heart diseases.

Secondly, the context or the background where an envisaged home-based health care programme is to be implemented was also defined, considering the cultural and economic factors of poor living standards in rural areas of Namibia as characterized by low socio-economic background of households headed by an agricultural substance farmers that have a bearing on the implementation and applicability for a successful empowerment of these parents/caregivers (Dickoff, James, & Wiedenbach, 1964). To that end, the dynamics or the energy sources for the activity to mitigate the negative experiences were identified and described, the purposes and the objectives for the home-based health care programme were described and the content for a home-based health care programme was developed accordingly.

## 3. The Dynamics, Objectives and the Activities for the Home-Based Health Care Programme

Four dynamics to mitigate the negative experiences and to facilitate coping with the demands of caring for the parents or caregivers of children with heart diseases which were identified are: the restoration of healthy emotions, restoration of productive social functioning and social relations, mitigation of the effects of financial burden and financial difficulties and the restoration of optimal functional status for the children. Correspondingly, the specific objectives of the envisaged home-based health care programme are to facilitate emotional focused coping methods for the parents/caregivers and the children alike, facilitate problem-management or problem-focused coping by the parents/caregivers, and to facilitate the optimal physical functional status for the children.

**Dynamic 1: Restoration of experiences of healthy emotions for the parents/caregivers and the children with heart diseases** is focused on mitigating the experiences of shock, disbelief, sadness, fear and self-blame among the parents/caregivers and depression and anxiety by the children with heart diseases and the mitigation of self-inadequacy, helplessness & hopelessness to care for the child with a heart disease by the parents/caregivers (Higgison & Gao, 2008; Ulvik, Hanestad, Wentzel-Larsen, & Wahl, 2008; Van Tilburg, Chitkara, Palson, Levy, & Whitehead, 2008; Shu-Fan et al., 2007; Sayers, 2008).

The emotional negative experiences are all the manifestations of the upset of balance and mal-adaptive responses to the changes and the demands for caring which require counteractions to restore the balance. In this regards, it is essential that the parents/caregivers receive counseling for them to strike a balance between acknowledging the child's illness, the demands for care and the use of methods of coping, cherish each day they have with the child and not to be discouraged by the reality that lay ahead. Moreover, the employment of emotional control is more likely to translate into the child's control over the symptoms, able to see the bright side of the situation and not always take their situation seriously (Stajduhar, Leigh Martin, Barwish, & Fyle, 2008; Van Tilburg et al., 2008; Shu-Fan et al., 2007).

Therefore, the interventions of the home-based health care programme about bereavement counseling, the use of emotional regulation techniques and meaning-based coping techniques, such as, positive appraisals & reinterpretation and the inculcation of optimism and self-efficacy can mitigate the impacts of the negative emotional experiences and facilitate coping (Cox et al., 2009; Ågren, 2008; Van Tilburg et al., 2008; Glanz et al., 2008; Brosig, Pierucci, & Leuthner, 2007; WHO, 2002; Egan, 1998).

Furthermore, these parents and caregivers are also expected to provide most of the instrumental task of caring, manage complex symptoms at home and to organize and coordinate health care activities on behalf of the child. If they do not have adequate knowledge & skills, they would be unsure of their ability to provide the necessary care at home. Therefore in addition to the interventions that are aimed at the restoration of healthy emotions, the parents/caregivers ought to be provided with health education about the child's illness, aspects of care and understanding the implications of compliance with the treatment for them to be able to reflect on or interpret the child's responses from the treatment (Paul, 2008; Strömbeg, 2005). To that end, it is important that the parents/caregivers of children with heart diseases in rural areas should get not only information for knowledge and training for skills, but their knowledge-based empowerment should include providing them with skills on how to promptly access help from the professional or community-based resource or person(s) if need be, in order to enhance safer care for their children and for them to cope with their caring journey (Glanz et al., 2008; Brosig et al., 2007).

**Figure 1:**
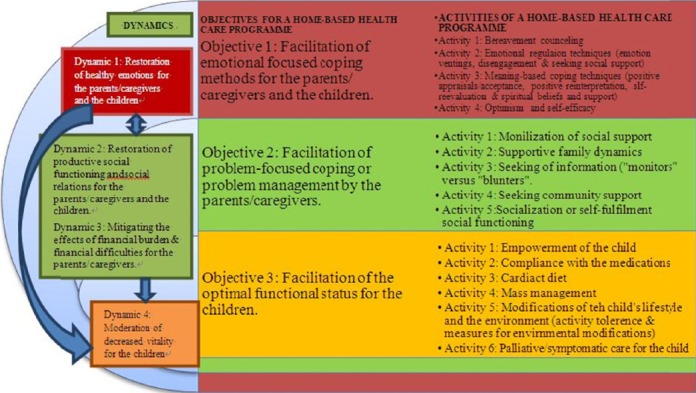
The conceptual framework for a home-based health care programme to facilitate coping with the demands of care by the parents/caregivers

If able to access information pertaining to their children's illness, the parents/caregivers in rural areas would be more prepared for what they may encounter during the course of care. Knowledge can therefore ease the burden of providing care as knowledge increases perceived control and therefore can facilitate the caregivers’ adaptation to the demands of care (Stajduhar et al., 2008; Strömbeg, 2005). Figure 1 illustrates the essence of knowledge to the improvement of the child's health status as a beneficiary of the caregivers’ empowerment.

**Figure 2:**
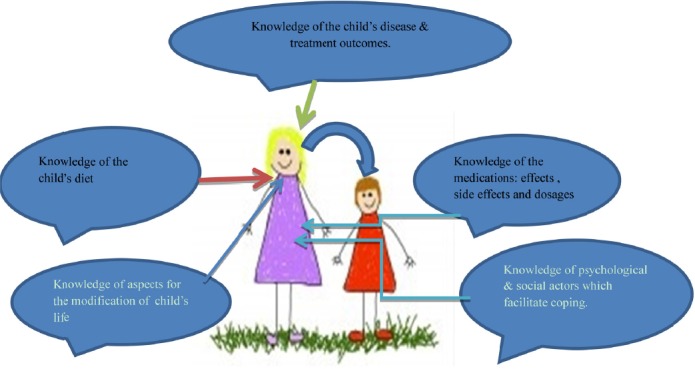
Knowledge & skills as enabling factors for the caregivers (Sourced from the www.shutterstock.com Accessed February 22, 2012)

A parents/caregiver who possesses appropriate knowledge and skills in return provides safer care to the child

**Figure 3:**
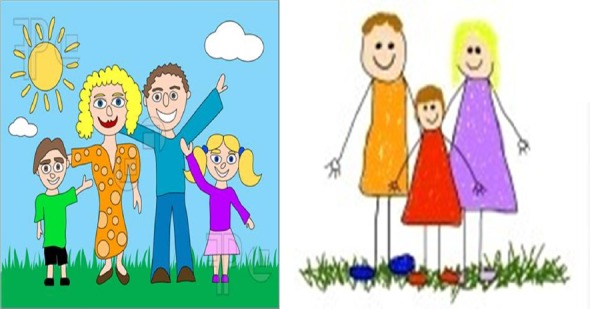
Supportive family dynamic (Sourced from the www.shutterstock.com Accessed February 22, 2012)

**Dynamic 2: Restoration of productive social functioning and social relations for the parents/caregivers and the children** should focus on the promotion of the supportive family dynamics, facilitation of productive social relations, social functioning, and facilitation of support from the societal organizations (Anderson-Moore, Whitney, & Kinukawa, 2009; Walsh, 2006; Krysan, More, & Zill, 1990). Supportive family dynamics are the family strengths that enable the family to deal with a crisis in a constructive manner and as a result, to realize growth out of such crisis and the challenging situations (Anderson-Moore et al., 2009; Walsh 2009; Krysan et al., 1990). Figure 3 presents the concept of effective family dynamics.

A focal caregiver who taps into supportive family dynamics can cope with the demands of care and therefore provides adequate and safe care that enables quality of life for the child. In addition, the siblings who can tolerate the sick child's limitations can help the sick child to build on his success and self-pride without the frustration of trying to force him/her performing strenuous physical activities which can compromise health. The ultimate outcome is a healthy, supported child as a beneficiary from the caregiver's empowerment.

In addition, the productive social relations, social support and social functioning can facilitate integration in the community and a close personal relationship with others, an effort that is a pathway for the parents/caregivers to access resources to facilitate coping with the demands to care for their children concerned (Berkman, 1995). To that purpose, and within their cultural context, the parents/caregivers should be urged to make use of guidance from the health care providers as intellectual resources about health related knowledge and skills that are necessary for safe performance of the instrumental tasks of care at home. Parents and caregivers should further make use of their confidant, community-based support groups who can provide them with social support. Other potential caregivers outside the family circle whose support the parents/caregivers of the children with heart diseases in rural areas can make use of are: friend, who can be relied upon for short-term minor care, and social workers to facilitate counseling and referential support. School personnel may also support the child during school hours as long as their involvement with the child's illness does not border to negligence of privacy and confidentiality or institute a ground for isolation and even dehumanization of the child at school (Webb, 2009; Major, 2003). This kind of support as based on one's area of confidence can help the parents/caregivers to get over with the negative experiences around their children's condition and to cope with the demands of providing care at home.

**Figure 4:**
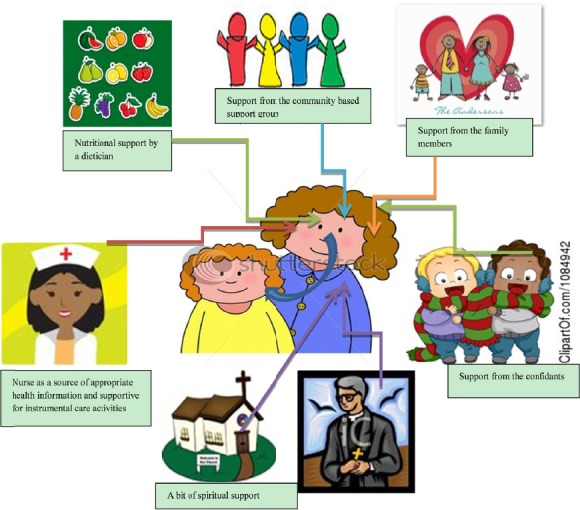
Mobilization of social support to facilitate coping with the demands of care (Sourced from the www.shutterstock.com Accessed February 22, 2012)

It can therefore be concluded that in order to achieve the restoration of caregivers’ productive social functioning, and promotion of their own well-being, a balanced coping can be solidified through the use of resources available at the family and community level (Major, 2003). Figure 4 presents social relations which can promote productive social functioning and coping for these parents/caregivers.

A health promotion approach characterized of multi-component interventions which draw all the determinant factors for coping (such as supportive family dynamics, confidants & downward comparisons, community support groups, health care providers, material resources and spiritual support) together can facilitate coping for the caregiver. In return, the caregivers provide quality care for the child with a heart disease.

**Dynamic 3: Mitigation of the effects of financial burden and financial difficulties for the parents/caregivers** should focused on facilitation of access to financial resources for the provision of special diet for the child and to cover the expenses related to traveling to the hospital for treatment purposes (Arafa, Zaher, EI-Dowaty, & Moneeb, 2008; Hill, Yucel, & Perrin, 2005; Beck & Wiencek-Kurek, 2007). Financial resources are the facilitative factors in health care services. The parents/caregivers of children, with heart disease who are from the rural areas in Namibia, need financial means to acquire an ideal diet for their children, as cardiac patients and to afford traveling to the hospital for the child's follow up treatment. To actualize this dynamic, it is important for these parents/caregivers to obtain some form of assistance to alleviate financial burden so they can cope with the demands of care at home. Although the facilitation for the provision of financial assistance to the parents/caregivers is not the domain of decision making for the individual health care provider's, a recommendations can be made for the children with heart diseases who are from economically vulnerable families to receive a social well-fare from the government to enable them to provide for the needs of care for these children.

**Dynamic 4: The moderation for the decreased vitality of the children** is focused on the empowerment of the child and moderation of experiences of physical dysfunctions (LeBlanck, Goldsmith, & Patel, 2003; Coovadia & Wittenberg, 2007; Mogotlane, Mokoena, & Chauke, 2005). In order to realize this dynamic, these children ought to be empowered for them to challenge their experiences, to demystify their negative assumptions about the course of their illness and potential treatment outcomes, but rather be encouraged to make adjustments to the course of the illness, thereby gaining control over their lives (Page & Czuba, 1999). Adjusting to a chronic illness can provide an excellent opportunity for a child to master crucial skills and prompts strong self-esteem and confidence. Therefore, the children can be in the position to minimize the negative perceptions and behavioral aspects related to their experiences of symptom burdens of heart disease. Psychosocial adjustment of these children as patients engenders the adherence to treatment regimens, thus allowing these children to play a crucial role in promotion of their vitality, early identification of illness-related setback, and early interventions for prevention. As a result of empowerment, these children would be able to adjust to the course of their illness, the results of which can enhance coping with the demands of care by their parents and caregivers (LeBlanck, Goldsmith, & Patel, 2003).

Other aspects which influence the children's vitality and therefore which need to be addressed are: adherence to the medications, cardiac diet and nutrition, management of the child's body weight, modification of child's lifestyles, as well as the modification of the environment. It is therefore indicated that caregivers be knowledgeable and skillful about aspects of medications such as dosages and side effects and to seek the assistance of a health care provider should the child experience side-effects from the medications (Higgins, Thoebald, & Peter, 2008; Reinhard, 2008; Grubb & Newy, 2006; Travis, Bethia, & Winn, 2000; Scherbring, 2002; Bucher et al., 2001; Schumacher, Stewart, Archbold, Dodd, & Dibble, 2000).

Although these parents/caregivers are from poor households which are food-unsecured and therefore their limited capacity to improve food choices for the children concerned, the view of Selektor et al. (Selektor & Weber, 2008; Charlton & Jooste, 2001) is adopted to focus the nutritional education for these caregivers on food that are available, affordable, and popular as long as they are consistent with the low-salt, low-fat and high-fiber formula for cardiac patients, while rich in vitamins to compensate for the low metabolism that results from the slow circulation as characterized by failure to thrive among these children (Chummun, Gospaul, & Lutchman, 2009; Weber, 2008; Ford-Martin, 2006; Menon & Poskitt, 1985).

As a method of life modification for these children, the literature suggests that the parents/caregivers should allow the children to perform those activities that are allowed by the medical personnel (Sayers, Riegel, Pawlowksi, Coyne, & Samaha, 2008; Jollife et al., 2001) and therefore tolerable to the child and, that should the child experience the signs of activity intolerance such as tiredness and respiratory distress, the child should preferably resume bed rest in order to reduce the heart's workload and the demand for oxygen (Sue, 2011; Eshah & Bond, 2009).

In addition, the parents/caregivers ought to be well-informed that polluted air poses the risk to airways infection and these complications tend to worsen the heart disease. As such, they need to maintain environmental hygiene in their houses and to prevent the child from exposure to second hand smoking. Practicing outdoor smoking by family members who smoke is therefore recommendable. Furthermore, as these families (from the rural areas of Namibia) make use of open fire to cook and (heat up in winter), it is important to advice the parents/caregivers to prevent the child from exposure to smokes and heat from open fires (Paul, 2008; Menon & Poskitt, 1985).

In conclusion, aspects of symptomatic care which involves providing basic comfort measures and which include bathing, ventilation, positioning and relieving of chest pain as well as recognizing the intensification of symptoms, a decision to seek services of a health care provider and the management of follow up should be explained to the parents/caregivers. Practice of oral hygiene and provision of immune boosting diet and protection of the child from illnesses that are associated with heart diseases, such common cold and as respiratory infections are to be clarified to the parents/caregivers (Coovadia & Wittenberg, 2007; WHO, 2002).

As borrowed from the concept of “dying person's Bill of Right”, through the programme interventions, the beneficiaries’ emotional, social and functional challenges would be met. In particular, the parents and caregivers would be able to cope and in return, the children would receive quality care, are allowed to retain a sense of hope and as a result, their health status would improve (Forrester, 2008).

The incorporation of Dickoff et al. (1964)'s element of the “goal content and procedure” to the content for a home-based health care programme displayed in Figure 1.

The conceptual framework for a home-based health care programme explains a complimentary relationship of problem-management and emotional coping. In return, a healthy emotional status of the caregiver influences the child's perception of coping with the burden of the diseases. Furthermore, the ability to manage the problems enables the caregivers to provide quality care that in return facilitates optimal functional status for the children, hence the parents/caregivers’ coping with the demands of care.

## 4. Discussion

The implementation of activities that are aimed at facilitating the emotional focused coping methods can enhance restorations of healthy emotions for the parents/caregivers and the children alike. Likewise, the implementation of activities which are aimed at facilitation of problem focused coping enables the participants’ access to social network and social resources, restoration of productive social functioning and –relations, and the mitigation of financial burden and difficulties respectively. The result would be that, the parents/caregivers and their children concerned are able to cope with their situation at home.

## 5. Conclusions

Effective mitigation of the negative experiences and facilitation of coping with the demands of care for the parents/caregivers of children with heart disease who are from the poor socio-economic background, requires the implementation of a home-based health care programme of multi-component interventions, which draws together all the possible determinants factors and the coping methods to the parents/caregivers and their children with heart disease for them to implement self-care goal directed activities to facilitate coping with the demands of caring at home. As a function of the implementation of the interventions of the envisaged home-based health care programme, three main coping outcomes, namely the emotional well-being for both the parents/caregivers and the child, and the problem management by the parents/caregivers as the recipients of the programme interventions, as well as the attainments of optimal functional status of the children as the beneficiaries of their parents/caregivers’ coping efforts would be attained (Glanz et al., 2008).
